# Changes of Intracellular Porphyrin, Reactive Oxygen Species, and Fatty Acids Profiles During Inactivation of Methicillin-Resistant *Staphylococcus aureus* by Antimicrobial Blue Light

**DOI:** 10.3389/fphys.2018.01658

**Published:** 2018-11-28

**Authors:** Jiaxin Wu, Zhaojuan Chu, Zheng Ruan, Xiaoyuan Wang, Tianhong Dai, Xiaoqing Hu

**Affiliations:** ^1^State Key Laboratory of Food Science and Technology, Jiangnan University, Wuxi, China; ^2^State Key Laboratory of Food Science and Technology, School of Food Science and Technology, Nanchang University, Nanchang, China; ^3^International Joint Laboratory on Food Safety, Jiangnan University, Wuxi, China; ^4^Department of Dermatology, Harvard Medical School, Boston, MA, United States

**Keywords:** antimicrobial blue light, methicillin-resistant *Staphylococcus aureus*, coproporphyrin, unsaturated fatty acids, membrane injuries, lipids

## Abstract

Antimicrobial blue light (aBL) has attracted increasing interest for its antimicrobial properties. However, the underlying bactericidal mechanism has not yet been verified. One hypothesis is that aBL causes the excitation of intracellular chromophores; leading to the generation of reactive oxygen species (ROS) and the resultant oxidization of various biomolecules. Thus, monitoring the levels of redox-sensitive intracellular biomolecules such as coproporphyrins, as well as singlet oxygen and various ROS may help to uncover the physiological changes induced by aBL and aid in establishing the underlying mechanism of action. Furthermore, the identification of novel targets of ROS, such as fatty acids, is of potential significance from a therapeutic perspective. In this study, we sought to investigate the molecular impact of aBL treatment on methicillin-resistant *Staphylococcus aureus* (MRSA). The results showed that aBL (5–80 J/cm^2^) exhibited a bactericidal effect on MRSA, and almost no bacteria survived when 80 J/cm^2^ had been delivered. Further studies revealed that the concentrations of certain intracellular molecules varied in response to aBL irradiation. Coproporphyrin levels were found to decrease gradually, while ROS levels increased rapidly. Moreover, imaging revealed the emergence and increase of singlet oxygen molecules. Concomitantly, the lipid peroxidation product malondialdehyde (MDA) increased in abundance and intracellular K^+^ leakage was observed, indicating permeability of the cell membrane. Atomic force microscopy showed that the cell surface exhibited a coarse appearance. Finally, fatty acid profiles at different illumination levels were monitored by GC-MS. The relative amounts of three unsaturated fatty acids (C_16:1_, C_20:1_, and C_20:4_) were decreased in response to aBL irradiation, which likely played a key role in the aforementioned membrane injuries. Collectively, these data suggest that the cell membrane is a major target of ROS during aBL irradiation, causing alterations to membrane lipid profiles, and in particular to the unsaturated fatty acid component.

## Introduction

As a novel light-based disinfection approach, antimicrobial blue light (aBL), particularly in the wavelength range of 405–470 nm, has attracted increasing interest due to its intrinsic antimicrobial effect. Compared to traditional photodynamic therapy, aBL therapy excites the endogenous chromophores of bacteria, and thus does not require the addition of exogenous photosensitizers. Furthermore, in comparison to ultraviolet irradiation, aBL shows much less detrimental effects in mammalian cells. The bactericidal activity of aBL is non-specific, and many microbial cells, including various antibiotics resistant strains, are highly sensitive to this treatment. aBL therapy has previously shown promise as a treatment for various clinical pathogens, such as *Pseudomonas aeruginosa*, *Acinetobacter baumannii*, methicillin-resistant *Staphylococcus aureus* (MRSA), and *Candida albicans* (Dai et al., 2012). In the case of *S. aureus*, the bactericidal effect has been reported to be highly efficient. For example, the survival rate of US300 MRSA (5 × 10^6^ CFU/ml cultures) was reduced by 96% under 405 nm aBL irradiation at 60 J/cm^2^ (Bumah et al., 2013), and survival fraction of MRSA USA300 (7 × 10^6^ CFU/ml cultures) was decreased by 98% under irradiation at 60 J/cm^2^ (Bumah et al., 2015).

However, the detailed mechanism by which aBL acts remains elusive. One hypothesis is that aBL excites photon-absorbing intracellular chromophores such as porphyrin, leading to energy transfer and the generation of reactive oxygen species (ROS), most notably singlet oxygen (^1^O_2_) (Hamblin et al., 2005). In such a scenario, the highly cytotoxic ROS may attack and oxidize various biomolecules nearby; including DNA, RNA and proteins (Kim and Yuk, 2017). Finally, this accumulation of oxidative damage would lead to bacterial death. However, up to now, the full details of the mechanism by which aBL acts are still not understood (Wang et al., 2017). Furthermore, potential novel targets of ROS, such as outer membrane fatty acids, have not been defined. In the current study, the effect of sub-lethal aBL irradiation on MRSA252 was studied in detail, and the levels of intracellular coproporphyrin, ^1^O_2_ and ROS were monitored.

Singlet oxygen has a short lifetime, usually microseconds within cells, and thus it is difficult to detect it directly. However, an indirect method for measuring ^1^O_2_ was previously developed by employing singlet oxygen sensor green (SOSG), a reagent which is highly selective for ^1^O_2_. SOSG is liable to be oxidized by ^1^O_2_ to form its endoperoxide, which emits a green fluorescence with an excitation peak at 504 nm and an emission peak at 525 nm (Flors et al., 2006). Until now, however, the SOSG-based ^1^O_2_ imaging method has only been applied in mammalian cells (Gollmer et al., 2011; Rac et al., 2015). Thus, the procedure was modified in the present study for use in bacterial cells. MDA (the oxidized product of lipids) was also quantified and monitored during sublethal aBL illumination, in order to assess oxidative stress.

It is known that ROS can result in cell injuries, including cell death, through the oxidation of biological molecules. The oxidation of thiol-containing proteins (Tardu et al., 2017) and nucleic acids such as DNA (Bumah et al., 2015) leads to the inactivation of enzymes and causes DNA to assume different conformations, ultimately resulting in cell death (Bumah et al., 2017; Tardu et al., 2017). Other biomolecules, such as lipids – which are mainly embedded within the cell membrane – are known to be susceptible to oxidation, but the downstream effects of such oxidation have not been as well-studied. Therefore, the influence of aBL irradiation on fatty acid profiles, including composition and relative content, were investigated in the current study by GC-MS.

Since lipids and proteins are important building blocks of cell membranes, damage to these biomolecules may lead to visible injuries on the cell surface. Thus, we also investigated changes in the morphology of the cell surface. Atomic force microscopy was employed to assess surface roughness and flame atomic absorption spectrometry was used to measure the leakage of K^+^ ions outside of cells (Ioannou et al., 2007).

The overall aim of the present study was to investigate the bactericidal mechanism of aBL and to identify novel target biomolecules of ROS during aBL irradiation.

## Materials and Methods

### Bacterial Strain and Growth Conditions

MRSA252 was grown on LB agar plates (10 g/L tryptone, 5 g/L yeast extract, 10 g/L NaCl, and agar 15 g/L, and pH controlled at 7) for 24–48 h. Then a single colony was picked up and incubated to 10 mL LB media without agar at 200 rpm for 5–10 h, until the optical density at 600 nm (OD_600_) was increased from the initial 0.02 to 1. Both incubations were carried out at 37°C.

### aBL Irradiation

To prepare the cell suspension, 3 mL of culture broth at an OD_600_ of one was firstly centrifuged at 8000× *g* and 4°C for 5 min, and the pellets were re-suspended in 6 mL sodium phosphate buffer (PBS) at 0.2 M and pH 7.0. After shaking for 3 min, the cell suspension was centrifuged again, and re-suspended in 2 mL PBS. The washing with PBS was repeated for three times, and the OD_600_ of the cell suspension was kept at 0.5 (Lipovsky et al., 2009).

Later, aBL illumination was conducted as follows. 5 mL of cell suspension at an OD_600_ of 0.5 was transferred to one well of a 6-well Clear Flat Bottom TC-treated Multiwell Plate, with a diameter at 36 mm and a height of 16.5 mm. The 6-Well Plate was placed on a magnetic stirring apparatus and gently stirred by a mini-magnetic bar (Fisher Scientific Co., Norcross, GA, United States) at 30 rpm. The blue light source was from the Omnilux clear-UTM light emitting diode (LED) array (Photo Therapeutics, Inc., Carlsbad, CA, United States), with a central wavelength of 415-nm and a full-width half maximum of 20 nm. The LED array aperture was fixed by an iron stand above the cell suspension and the distance to the surface of the cell suspension was adjusted to achieve an irradiation level of 16.7 mW/cm^2^ (i.e., 1 J/cm^2^ per min), which was detected by a PM100D power meter.

To avoid the interference of sunlight, the sides of the 6-well Plate, except the top and bottom, were sealed by tin-foil. For the control, all six sides were covered. To avoid cell death due to a rise in temperature during aBL illumination, an air cooler was used to keep the ambient air temperature below 20°C.

To measure the bacterial population, 20 μL of the cell suspensions at 0, 10, 20, 30, 60, 120, and 240 min, corresponding to irradiation doses of 0, 10, 20, 30, 60, 120, and 240 J/cm^2^, were sampled. After serial dilutions, the cell density was assayed by the colony-count technique, and the survival rate was calculated.

To analyze a series of biological changes under sub-lethal conditions, such as intracellular K^+^ leakage, ^1^O_2_, ROS, and fatty acid profiles, the cell suspensions at sub-lethal aBL doses of 1, 2, 3, 4, 5, and 6 J/cm^2^ were sampled, respectively, and the measurements were conducted as outlined below.

### Determination of Intracellular Coproporphyrin Content

*Staphylococcus aureus* suspensions were centrifuged at 6,000× *g* and 4°C for 10 min, and the pellets were re-suspended in 0.1 M NH_4_OH-acetone solution (1:9, v:v) (Braatsch and Klug, 2004; Kossakowska et al., 2013) in a dark environment. After 24 h, the solution was centrifuged at 6,000× *g* and 4°C for 10 min, and coproporphyrin in the supernatant was detected employing the HPLC method (Nitzan et al., 2004).

### Assays of ^1^O_2_ and ROS

Singlet oxygen was bound by the SOSG reagent and imaged by confocal laser scanning microscopy (CLSM), according to the method previously described (Rac et al., 2015), with minor modifications as follows. Firstly, 400 μL methanol solution of SOSG at 500 μM was mixed with 3.6 mL of bacterial suspension at a cell density of 10^8^ CFU/mL in the 35-mm petri dish mentioned above. Next, samples with and without aBL irradiation were prepared separately in single concave slides (Sail Brand, Jinliu Instrument Co., Ltd., Nanjing, China) covered with nail oil to prevent water evaporation. Finally, the CLSM images were obtained by sequential scan (TCS SP8, Leica Microsystems Inc., Heidelberg, Germany) with an excitation wavelength at 488 nm and an emission wavelength at 525 nm.

To detect intracellular ROS, a fluorescent probe 2′,7′-dichlorofluorescin diacetate (DCFH-DA) was employed. DCFH-DA reacts with ROS to form the fluorescent DCF, which was detected using a spectrofluorophotometer with excitation wavelength at 485 nm and emission wavelength at 525 nm. The cell suspension was centrifuged immediately at 8000× *g* and 4°C for 5 min, and the bacterial precipitate was washed twice with PBS, as mentioned above. The resultant bacterial suspension at OD_600_ at 0.5 was incubated with DCFH-DA (10 mmol/L) at room temperature for 20 min in the dark. The mixture was then centrifuged at 4°C and 8000× *g* for 5 min, and the bacterial precipitate was washed twice with PBS at 4°C, in order to remove the DCFH-DA that had not entered the cell but attached to the cell surface. Finally, the intracellular ROS content was analyzed using a spectrofluorophotometer following the protocol of ROS assay kit (Kit # S0033, Beyotime Biotech, China).

### Quantification of Lipid Peroxidation

Reactive oxygen species cause lipid peroxidation process in many organisms. Malondialdehyde (MDA) is one of the terminal products of lipid peroxidation in cells, and commonly regarded as a marker of oxidative stress. In the present study, thiobarbituric acid was used to react with MDA to form an adduct that could be quantified colorimetrically at 532 nm. Firstly, 1 mL of cell suspension was collected by centrifugation and re-suspended in 1 mL PBS. The cell suspension was quickly immersed in liquid nitrogen for 2 min, then taken out and laid on ice. After thawing, the cell suspension was centrifuged at 4°C and 8,000× *g* for 5 min. Finally, 150 μL of supernatant was pipetted into a 1.5 mL cuvette to detect the MDA content.

Malondialdehyde was analyzed by a colorimetrical method as follows. Firstly, 150 μL of the supernatant was mixed with 300 μL of MDA working solution. Next, the reaction mixture was heated at 100°C for 25 min, then cooled to room temperature, and centrifuged at 4°C and 6,000× *g* for 15 min. Finally, the absorbance at 532 nm of the supernatant was determined and the MDA content was calculated based on a standard curve. The detailed procedure was described in the protocol of Beyotime of China (Kit #S0103, Beyotime, China).

### Extraction and Analysis of Fatty Acids From Sub-Lethal Bacteria

Fatty acids were extracted according to the previously described method (Li et al., 2010; Wang et al., 2016), with some minor modifications as follows: (1) 1 mL cell suspensions were centrifugated at 4°C and 6,000× *g* for 10 min; (2) the pellets were immersed in liquid nitrogen for 3 min; (3) the quenched cells were resuspended in 1 mL of NaOH solution at 4 M, and incubated at 90°C for 120 min; (4) the pH of the cell lysate was adjusted to 3.0 by HCl; (5) after the cell lysate was cooled to room temperature, 2 mL of anhydrous diethyl ether was added and incubated at 37°C for 8 h; (6) the extract liquid was centrifugated at 6,000× *g* for 10 min, the upper phase was pipetted smoothly, and 0.2 g anhydrous sodium sulfate was added; (7) 12 h later, the ether phase was pipetted smoothly, then dried in a stream of nitrogen, and dissolved in 50 μL hexane; (8) 25 μL of bistrimethylsilyl trifluoroacetamide (BSTFA) was added, and incubated at 50°C for 30 min; (9) GC-MS analysis was carried out based on the previously described method (Li et al., 2010).

### Observation of Surface Morphology by AFM

Firstly, 500 μL of the unirradiated and irradiated cell suspensions were centrifuged at 6,000× *g* and 4°C for 10 min. The precipitate was re-suspended in 500 μL sterile water and centrifuged again. Next, the pellet was re-suspended in 500 μL sterile water and mixed with 5% glutaraldehyde (TAAB Laboratories, United Kingdom) at 4°C. 12 h later, the cell suspension was centrifuged and the pellet was re-suspended in 100 μL sterile water. Finally, 5 μL of bacterial suspension was dripped onto glass slides, and dried at room temperature overnight for AFM observation (Anderson et al., 2004; Merghni et al., 2016).

### Detection of K^+^ Permeability of the Outer Membrane

To detect the permeability of outer membrane, the level of extracellular K^+^ ion was measured according to the previous reports (Ioannou et al., 2007; Ding et al., 2016), with some minor modifications as follows. The cell suspension was centrifuged at 4°C and 8,000× *g* for 5 min, and the supernatant was obtained with a pipette. K^+^ concentration in the supernatants was determined using flame atomic absorption spectrometry at 766.5 nm. The instrument was calibrated by employing K^+^ standards at 0.1, 0.2, 0.4, 0.8, 1.6, and 3.2 mg/L. Since there was a linear relationship between emission and potassium concentration, K^+^ was assayed by standard curve and the leakage rate was calculated by the formulation (Ioannou et al., 2007).

### Statistical Analysis

For the experiments mentioned above, three independent trials were performed in order to obtain the mean values, and the values were expressed as mean ± standard deviation. Differences in the values of the untreated control and the irradiated groups were compared for statistical significance using one-way ANOVA, and *p*-values of <0.05 were considered significant.

## Results

### aBL Inactivation of MRSA

**FIGURE 1:**
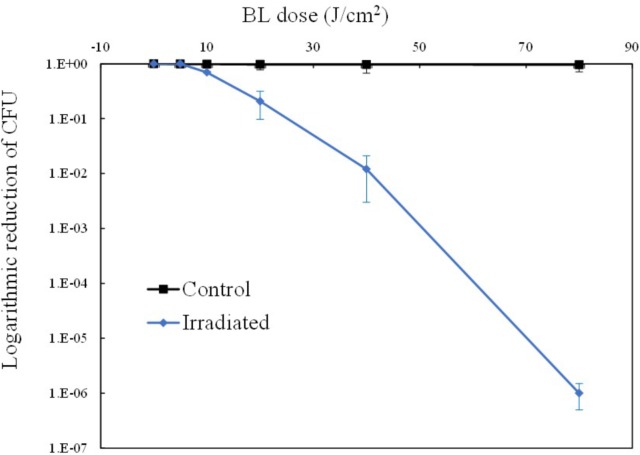
Light dose-dependent photodynamic inactivation of MRSA252 *in vitro*. Experiments were performed in triplicate. Data are means, and bars represent standard deviations.

A 415-nm LED was employed to illuminate the suspension of MRSA 252 cells. As shown in Figure 1, the irradiated MRSA cells displayed a decreasing survival curve, indicating that the bactericidal effect of aBL is dose-dependent, whereas the unirradiated cells showed a stable unchanged curve. It showed that 0–5 J/cm^2^ was a sub-lethal dosage, since almost no reduction in cell survival occurred. Thereafter, MRSA was inactivated gradually, and the bactericidal curve approximately followed first-order kinetics. When 80 J/cm^2^ was delivered, a significant reduction above 6 log_10_ CFU was achieved (*P* < 0.01).

It was of significance to examine the changes in the levels of intracellular molecules such as coproporphyrin, ROS and lipid oxides, which are thought to be critical for the mechanism of action of aBL inactivation. Thus, these analyses were performed under sub-lethal aBL doses and the results are outlined below.

### Changes in the Level of Intracellular Coproporphyrin

**FIGURE 2:**
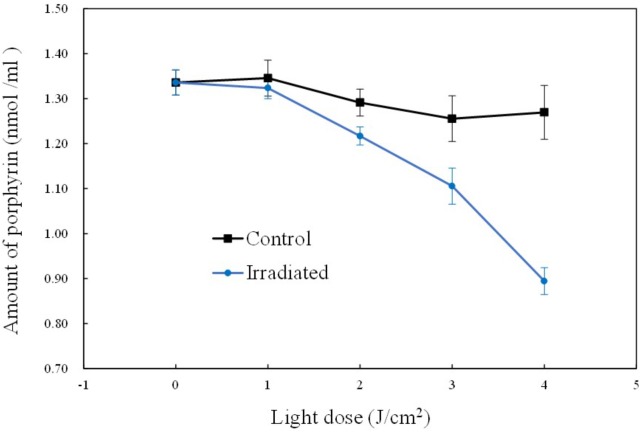
Curves of the intracellular coproporphyrin levels during sub-lethal irradiation of MRSA252. Data are means, and bars represent standard deviations.

In the commonly accepted hypothesis, endogenous porphyrins are excited by aBL, which is thought to be critical for the generation of ROS and subsequent ROS-mediated damage to the bacterial cell. Thus, coproporphyrin, which is a highly abundant porphyrin in *S. aureus* (Nitzan et al., 2004) was monitored herein. Figure 2 shows the different patterns of intracellular coproporphyrin changes between the irradiated cells and unirradiated cells. Comparatively speaking, the concentration of coproporphyrin in the dark (control) remained stable, with a slight fluctuation within 5%. However, under aBL irradiation, coproporphyrin levels showed an obvious decrease of 33.70%. It dropped from the initial level of 1.33 ± 0.03 ppm at 0 J/cm^2^ to 0.89 ± 0.01 ppm at 5 J/cm^2^. The gradual reduction of coproporphyrin was likely due its consumption in a stepwise manner during aBL treatment (*P* < 0.05).

### Changes in Intracellular ^1^O_2_ and ROS Contents

**FIGURE 3:**
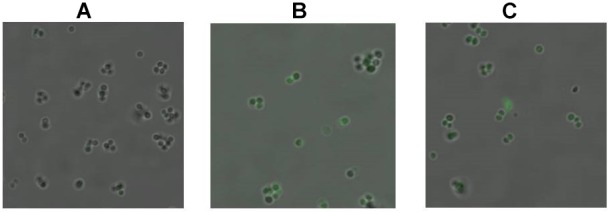
Detection of singlet oxygen with 0 J/cm^2^ **(A)**, 2 J/cm^2^ **(B)**, and 4 J/cm^2^ **(C)** dose of aBL irradiation by CLSM. The formation of ^1^O_2_ was measured in non-irradiated and irradiated MRSA252 cells using fluorescent probe SOSG.

Singlet oxygen may be generated immediately after the excitation of intracellular sensitive chromophores. ^1^O_2_ has a very short half-life, and therefore a fluorescent probe SOSG has previously been used as a commercial reagent for ^1^O_2_ imaging in mammal cells. In the present study, SOSG was also used for ^1^O_2_ imaging within MRSA cells by CLSM, with some modifications to the previously described approach. Figure 3 shows the fluorography of MRSA cells before and after aBL illumination, respectively. Non-irradiated cells did not exhibit any fluorescence even in the presence of SOSG. In contrast, the irradiated cells emitted increasing levels of green fluorescence, along with a concomitant decrease in coproporphyrin (Figure 2), indicating an inverse association of coproporphyrin and ^1^O_2_.

**FIGURE 4:**
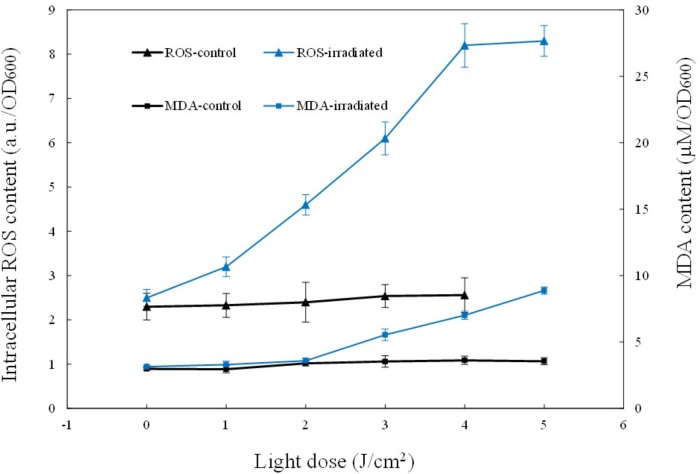
Curves of the intracellular ROS and MDA levels during sub-lethal irradiation of MRSA252. Data are means, and bars represent standard deviations.

Singlet oxygen is the primary product of the excitation of intracellular chromophores, and it could attack other molecules nearby to produce other ROS, such as peroxides, superoxide, and hydroxyl radical. Therefore, the total ROS content was also examined. As shown in Figure 4, for the control, almost no ROS fluctuation occurred. In comparison, the illuminated cells showed 3.6-fold elevation of ROS. The intracellular ROS level was 2.20 ± 0.19 a.u./OD_600_ before aBL treatment, and it ascended rapidly to 8.20 ± 0.21 a.u. at 4 J/cm^2^. The dynamic changes of coproporphyrin, ^1^O_2_ and ROS supported the excitation of chromophores and generation of ROS (*P* < 0.01).

### Changes in Intracellular MDA Content and Fatty Acid Profiles

Compared to ^1^O_2_, ROS has a longer half-life, and thus it has more chance to oxidize biomolecules nearby such as proteins or lipids. Hence, the oxidative stress induced by ROS was assessed by measuring MDA, a biomarker of lipid oxidation. In the current study, MDA was detected by the thiobarbituric acid-based colorimetric method. The results show that the irradiated cells exhibited a gradual increase in intracellular MDA content, coinciding with the increase of ROS (Figure 4). When 5 J/cm^2^ was delivered, the intracellular ROS level increased three-fold and meanwhile, the intracellular MDA content was increased 2.81-fold, from the initial 3.15 ± 0.19 μM/OD_600_ to the final 8.88 ± 0.35 μM/OD_600_. These data indicate that aBL-induced ROS induced increased MDA levels, and indicate that cell death was caused by the aggravation of oxidative stress within cells.

To further investigate the influence of ROS on lipids, fatty acid profiling was assayed using LC/MS. As shown in Table 1, there existed distinct differences in the composition and relative percentages of fatty acids between the unirradiated cells and irradiated cells. About 16 fatty acid species, including saturated and unsaturated fatty acids, were detected before aBL illumination, while the unsaturated fatty acid component (C_16:1_, C_20:1_, and C_20:4_) gradually disappeared in response to aBL irradiation. The initial levels of C_16:1_ and C_20:1_ were 0.08 ± 0.01 and 0.08 ± 0.03%, respectively, while both of these became non-detectable after treatment. Meanwhile, C_20:4_ decreased to 0.15 ± 0.02% at 1 J/cm^2^ and disappeared at 2 J/cm^2^ (Table 1). Comparatively speaking, there are no obvious changes in the saturated fatty acid profiles, and this may indicate that monounsaturated and polyunsaturated fatty acids are particularly susceptible to ROS generated upon aBL illumination. In general, the current data proved that unsaturated fatty acids are vulnerable targets of ROS during aBL inactivation (*P* < 0.05).

**Table 1:** The effects of different sub-lethal doses on fatty acid profiles in MRSA252.

Cellular fatty acids	Percentage (%)			
	0 J/cm^2^	1 J/cm^2^	2 J/cm^2^	4 J/cm^2^
C_12:0_	0.52 ± 0.04	0.49 ± 0.02	0.51 ± 0.03	0.53 ± 0.03
C_14:0_	1.63 ± 0.11	1.62 ± 0.08	1.59 ± 0.09	1.48 ± 0.08
isoC_15:0_	23.45 ± 2.23	23.27 ± 3.56	22.96 ± 2.78	20.65 ± 3.20
anteisoC_15:0_	27.88 ± 3.23	25.47 ± 1.96	26.55 ± 2.44	24.73 ± 3.05
C_15:0_	2.39 ± 0.14	2.48 ± 0.04	2.13 ± 0.05	2.01 ± 0.12
C_16:1_	0.08 ± 0.01	ND	ND	ND
C_16:0_	10.62 ± 0.51	8.75 ± 0.62	9.73 ± 0.39	8.06 ± 0.44
isoC_17:0_	4.71 ± 0.27	4.84 ± 0.36	3.96 ± 0.22	4.16 ± 0.17
anteisoC_17:0_	5.04 ± 0.46	4.89 ± 0.21	4.79 ± 0.19	4.83 ± 0.14
C_17:0_	0.65 ± 0.09	0.62 ± 0.07	0.58 ± 0.04	0.59 ± 0.04
C_18:0_	10.29 ± 1.18	10.31 ± 1.25	10.18 ± 1.02	10.04 ± 1.00
isoC_19:0_	1.96 ± 0.16	1.93 ± 0.13	1.89 ± 0.12	1.97 ± 0.19
anteisoC_19:0_	1.47 ± 0.08	1.38 ± 0.17	1.48 ± 0.13	1.22 ± 0.11
C_20:4_	0.29 ± 0.05	0.15 ± 0.02	ND	ND
C_20:1_	0.08 ± 0.03	ND	ND	ND
C_20:0_	2.68 ± 0.28	2.73 ± 0.30	2.67 ± 0.19	1.82 ± 0.22

*ND, not detectable*.

### Changes in Cell Surface Morphology

**FIGURE 5:**
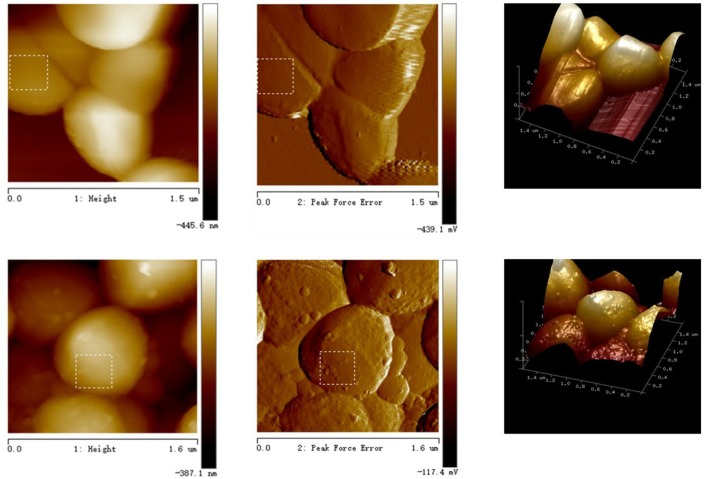
Observation of MRSA cells by atomic force microscope. Top: in the dark; bottom: under aBL irradiation.

Most bacterial lipids are located at the cell membrane, thus AFM imaging was employed to investigate whether marked alterations occurred in the cell surface upon aBL irradiation. As expected, a smooth surface morphology was observed for the unirradiated cell (Figure 5), whereas there were some granular bulges on the surface of aBL-treated cells. This phenomenon was supported by significant increases in two roughness parameters: Rq (root mean square of the heights) and Ra (average roughness). For the unirradiated cells, the Rq and Ra values of the selected area were 6.50 and 5.07 nm, respectively, while for the illuminated cells these values were 14.0 and 11.2 nm, respectively. In general, AFM micrographs revealed that most cells maintained intact morphologies, with marked roughness visible after aBL irradiation, perhaps due to the damage of biomolecules embedded in the cell surface, such as lipids and membrane proteins.

### Changes in Cell Membrane Permeability

**FIGURE 6:**
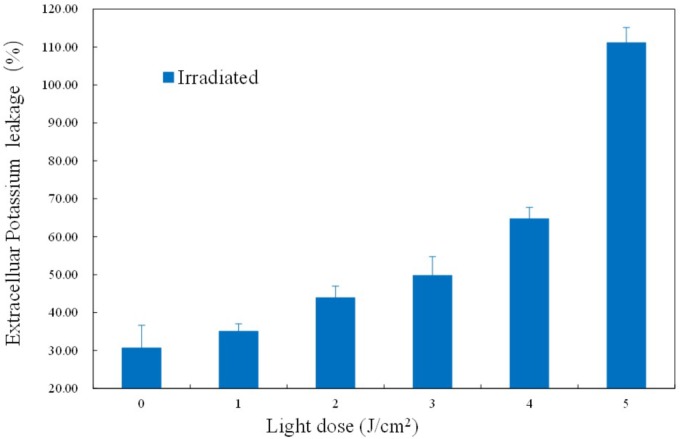
Effects of different aBL doses on the leakage rate of K^+^.

As an important physiological parameter, the permeability of the cell membrane is critical for substrate uptake and product secretion. Extracellular K^+^ concentration was analyzed by flame atomic absorption spectrometry and the K^+^ leakage rate was calculated. As shown in Figure 6, the K^+^ leakage started at 3 J/cm^2^ and increased gradually later, suggestive of an increasing and uncontrollable permeability. Under sub-lethal aBL does, more and more the transmembrane protein Na^+^-K^+^ pump was likely denatured, leading to a gradual reduction of K^+^ uptake. The damage and denaturation of other membrane proteins, as well as the oxidation of membrane lipids, might play a critical role in compromising cell membrane integrity (*P* < 0.05).

Generally speaking, the current study confirmed dynamic changes in the intracellular levels of coproporphyrin, ^1^O_2_ and ROS, and revealed unsaturated fatty acids as likely targets of ROS. The oxidation of lipids, as well as membrane proteins, may partly contribute to membrane injury and cell lysis. The current study supported the antimicrobial hypothesis of aBL (Hamblin et al., 2005), and the underlying mechanism was proved as follows. The aBL firstly excited the intracellular porphyrin, resulting in the generation of ^1^O_2_ and ROS. Later, the cytotoxic ROS oxidized various lipids, especially the unsaturated fatty acids. The disappearance of unsaturated fatty acids and alteration of fatty acid profiles led to cell membrane damage, such as leakage of intracellular components, and ultimately contributed to bacterial death.

## Discussion

Recently, more and more research has indicated that aBL is effective in the photoinactivation of antibiotic-resistant bacteria. aBL was similar to photodynamic therapy, while need not the addition of exogenous photosensitizers. Another advantage was that the susceptibility of the organism and antibiotics in *S. aureus* are barely affected after repeated exposure (Tomb et al., 2017).

However, the bactericidal mechanism of action of aBL is still not fully understood. Therefore, monitoring physiological changes under sub-lethal aBL irradiation is critical for the clarification of the underling mechanism. Here, we monitored a number of intracellular biomolecules, including coproporphyrin, ROS, MDA, and fatty acids, as well as the permeability of the cell membrane and morphology of the cell surface.

Endogenous coproporphyrin, a precursor of protoporphyrin IX producing the majority of free radicals (Nitzan et al., 2004), was first assayed. It was reported that the intracellular porphyrins seemed to be principally coproporphyrin (Amin et al., 2016), which was thus monitored during the process of aBL illumination in the current study. Amin et al. (2016) compared the HPLC profiles of porphyrins extracted from the unirradiated and irradiated *P. aeruginosa*, respectively, while did not take a quantitative analysis. Besides, there were few reports to identify and quantify the endogenous porphyrins. Unlike the previous publications, we quantitatively measured the endogenous coproporphyrin and revealed its dynamic change for the first time. During the process of aBL illumination, coproporphyrin levels decreased in a stepwise manner (Figure 2) in response to aBL treatment. Ghate et al. (2015) have previously assayed coproporphyrins levels by HPLC to understand the extent of their inactivation of the bacteria. Various strains were reported to show different susceptibilities to aBL, and this may be related to differences in the abundances of endogenous porphyrins. To identify the mechanism of the antimicrobial effects of blue light, future work could focus on the screening and identification of various endogenous photosensitizers of strains with different susceptibilities to aBL, perhaps using metabonomics.

In a biological context, ROS are regarded as natural byproducts of normal metabolism; however, their appearance in large numbers under environmental stress would lead to cellular structure (Vatansever et al., 2013). The influence on amino acid, DNA, and RNA integrity has been reported widely. As far as we know, however, there are few reports on ROS-mediated damage to bacterial lipids under aBL treatment. As shown in Figure 4, we found a rapid rise in MDA, indicating that lipids were indeed attacked by ROS. We analyzed fatty acid profiles and found a distinct alteration in composition and relative content. In total, 16 types of fatty acids were detected in MRSA 252, three of which disappeared in response to aBL treatment, including palmitoleic acid (C_16:1_), eicosenoic acid (C_20:1_), and arachidonic acid (C_20:4_). Compared to saturated fatty acids, the unsaturated fatty acids are vulnerable to oxidation. As expected, the saturated fatty acids profiles did not show obvious fluctuations during aBL irradiation (Table 1).

Since most lipids are located in the cell membrane, fatty acids are a major determinant of the biophysical properties of *S. aureus*; including its antimicrobial susceptibility, pathogenesis, and stress response (Sen et al., 2016). For the polyunsaturated fatty acids, the hydrogen atoms in the position of the methylene groups are especially reactive, which thus makes the methylene groups (-CH_2_-) between double bonds extremely vulnerable during ROS attack (Mylonas and Kouretas, 1999). Critical properties of fatty acids include chain length, saturation degree and relative content. 16- and 18-carbon fatty acids are common in bacteria, and these unsaturated fatty acids have important biological functions. Under certain conditions, the unsaturated/saturated fatty acid profile is important for bacterial membrane homeostasis (Parsons and Rock, 2013). Unsaturated lipids are mainly located in the cell membrane, and the peroxidation of these molecules could therefore change the composition of the cell surface and influence cellular morphology.

Evidence of damage to the cell membrane was found using atomic force microscopy (Figure 6). Irradiated MRSA cells under sub-lethal doses showed more roughness (Figure 5), an indicator of damage to the cell surface. The damage of cell membranes would likely be followed by leakage of cellular components and then by complete lysis of the cell, resulting in cell death.

The leakage rate of K^+^ was confirmed in the current study. K^+^, as well as Ca^2+^ and Mg^2+^, are stabilizers of the cell surface, and K^+^ is particularly essential for cell membrane permeability (Talaro and Talaro, 2002). In a previous report, ozone was found to cause non-selective permeability of the membrane, and facilitated K^+^ permeability (Zhang et al., 2011). Similarly, our results revealed increased K^+^ leakage (Figure 6), which may indicate denaturation of the transmembrane protein Na^+^-K^+^ pump.

Antimicrobial blue light exposure may induce damage to multiple targets within the cell membrane. In addition to lipids, membrane proteins are also regarded as important membrane building block, and endow cells with multiple functions such as transmembrane transport ability, adhesion, and information reception (Alves et al., 2013). The oxidation and denaturation of membrane proteins, therefore, would be expected to have a devastating effect on bacterial cell viability and may also play a role in the bactericidal effect of aBL.

In summary, the current study is, to our knowledge, the first demonstration of substantial quantitative evidence of changes in the fatty acid profiles of MRSA under aBL irradiation. We have confirmed some aspects of the bactericidal hypothesis, and provided some novel insights into the aBL inactivation mechanism. Future work will involve more detailed investigations of the dynamic lipidomic changes to fully understand the aBL inactivation process.

## Author Contributions

JW conducted most of the experiments and wrote the manuscript. ZC assayed ROS and MDA. XH and ZR designed the experiments and modified the paper. XW helped to reveal the membrane injuries. TD helped to construct the blue light device and verify the mechanism.

## Conflict of Interest Statement

The authors declare that the research was conducted in the absence of any commercial or financial relationships that could be construed as a potential conflict of interest. The reviewer YY declared a shared affiliation, with no collaboration, with one of the authors TD to the handling Editor at the time of review.
